# A Study of Parasitic and Bacterial Pathogens Associated with Diarrhea in HIV-Positive Patients

**DOI:** 10.7759/cureus.807

**Published:** 2016-09-27

**Authors:** Siddharth Shah, Vaishali Kongre, Varun Kumar, Renu Bharadwaj

**Affiliations:** 1 Department of Orthopaedics, Lokmanya Tilak Municipal Medical College & General Hospital, Sion, Mumbai; 2 Department of Microbiology, B. J. Government Medical College & Sassoon General Hospitals, Pune; 3 Department of Medicine, Shri Balaji Action Medical Institute

**Keywords:** diarrhea, infection, pathogens, parasites, bacteria, aids, hiv

## Abstract

**Introduction:**

Diarrhea is a common complication of acquired immune deficiency syndrome (AIDS), occurring in almost 90% of AIDS patients in developing countries like India. The present study was aimed to determine the prevalence and microbiological profile of pathogens associated with diarrhea in human immunodeficiency virus (HIV) positive patients and their relation to CD4 counts.

**Materials and methods:**

Forty-five successive HIV-positive patients, 27 with diarrhea (study group) and 18 without diarrhea (control group), were included in the three-month study. The HIV infection was confirmed by three different antibody detection tests. The stool samples were collected on two consecutive days and were examined for parasites by microscopy using wet mount and modified Ziehl-Neelsen stain. They were examined for bacteria by Gram stain and conventional Ziehl-Neelsen stain and were inoculated on appropriate culture media. The isolates were identified by standard biochemical tests, followed by antibiotic susceptibility testing using the Kirby-Bauer disc diffusion method.

**Results:**

Twenty-four pathogens were detected in diarrheal HIV-positive patients, including 14 parasites (58.33%), seven bacteria (29.17%), and three fungi (12.50%). *Isospora sp. *was the most common parasite (25.9%) followed by *Cryptosporidium sp. *(14.8%). Other parasites included *Cyclospora sp.*, *Strongyloides stercoralis,* and *Entamoeba histolytica* (3.7% each).​ *Escherichia coli* (18.5%) was the most common bacterial isolate, of which, 80% were *Enterotoxigenic E. coli* (ETEC) while 20% were *Enteropathogenic E. coli *(EPEC)*. *Other isolates included *Shigella flexneri* and *Mycobacterium tuberculosis *(3.7% each). The isolates were sensitive to furazolidone (94.11%), chloramphenicol (76.47%), and gentamicin (52.94%). The isolates from diarrheal patients showed resistance to norfloxacin (5.88% vs. 50%, p<0.05) as compared to those from non-diarrheal patients. The diarrheal HIV-positive patients had lower mean CD4 counts (202.6 cells/µL), as compared to those without diarrhea (239.28 cells/µL).

**Conclusion:**

*Isospora sp.* is the most common parasite and *Escherichia*​* coli* is the most common bacterium associated with diarrhea in HIV patients. The antibiotic sensitivity patterns should be monitored regularly to detect resistance to commonly used drugs. The prevalence of organisms in a region, various clinical manifestations, sensitivity patterns of isolates, and relation with CD4 count should be considered while instituting therapy in HIV patients with diarrhea.

## Introduction

With an adult prevalence of 0.26% (0.22%–0.32%) in 2015, human immunodeficiency virus (HIV) infection and acquired immune deficiency syndrome (AIDS) have become major health issues in recent times in India [1]. Since the first case of HIV/AIDS got diagnosed in 1986 from a commercial sex worker in Chennai, the number of people living with HIV (PLHIV) in India has gone up to 21.17 lacs in 2015, as per the data available from the National AIDS Control Organization (NACO) [1-2]. At the end of 2004, the world over, over 14,000 new infections were being acquired each day, and of these, more than 90% were in developing, low and middle-income countries including India [2].

Diarrhea is a common complication of AIDS. It occurs in almost 90% of AIDS patients in developing countries like India [3]. The World Health Organization (WHO) defines chronic unexplained diarrhea for more than one month as clinical stage III and chronic cryptosporidiosis or chronic isosporiasis with diarrhea as clinical stage IV in adults. [4]. When either of these conditions occur with a positive immunological test (CD4 count less than 350 cells/µL), it is a defining criterion for advanced HIV infection, including AIDS [4]. The infectious etiological agents include both opportunistic agents and non-opportunistic infections. The opportunistic agents cause severe, chronic or frequent gastrointestinal diseases, while non-opportunistic agents usually cause acute, treatable diarrheal illness. The management of diarrhea and its complications may require antibiotic therapy because antibiotics can shorten the duration of diarrhea, decrease stool output, and abrogate some complications. Due to the delayed diagnosis of HIV in these individuals, the patients usually take over-the-counter drugs or local medications for symptomatic relief and the underlying disease is left untreated. In a developing country like India, this often results in weight loss and wasting syndrome leading to profound morbidity.

In recent years numerous studies have outlined the emergence of important gastrointestinal protozoa like *microsporidia *species, *Cryptosporidium* species, *Isospora belli* and *Cyclospora cayetanensis *[5-6]. Amongst the HIV-positive patients, certain risk factors such as homosexuality and practicing oro-anal sex, can exacerbate the possibility of acquiring some intestinal parasitoses such as giardiasis, cryptosporidiosis, and strongyloidiasis, where symptomatic pictures are more serious than those of individuals with a non-compromised immune system. Intestinal parasitic diseases are the commonest and major cause of morbidity and mortality in HIV-positive individuals worldwide [6]. Various studies have outlined the bacterial etiologic agents for diarrhea in HIV-positive patients, which include *salmonella*, *shigella*, *campylobacter*, and *mycobacterium* species [7-8]. The treatment of bacterial diarrhea is further complicated by the emergence of resistance amongst the organisms to the commonly used antibiotics. Since the antibiotic susceptibility profiles vary from time to time and from region to region, there is a need for a periodic update of the susceptibility profiles and the prevalence of the enteric bacterial pathogens. The spectrum of opportunistic infections in HIV-infected patients varies from one region to another [9]. The proper identification of etiologic agents is fundamental for the clinical diagnosis, epidemiological study, prevention and control of diarrheal diseases in HIV-positive patients as well as to reduce the overall morbidity and mortality in these patients.

The pathogens infecting HIV-positive patients with diarrhea may differ genotypically and phenotypically from those in HIV-positive patients without diarrhea. Hence, a study of the organisms commonly found in the stools of non-diarrheal HIV-positive patients would reveal the prevalence of commensal organisms and provide the basis for comparing and analyzing the two groups for more effective results.

Thus, the present study was undertaken to determine the incidence and microbiological profile of enteric pathogens in HIV patients with and without diarrhea.

## Materials and methods

The study was conducted in the Department of Microbiology, B.J. Government Medical College (BJMC) and Sassoon General Hospitals, Pune, India, which is a tertiary care center. A total of 45 HIV-seropositive patients who were admitted to the general medicine or infectious diseases wards of Sassoon General Hospitals, were included in the study. Of the total patients included in the study, 27 presented with diarrhea as the sole or one of the chief complaints. Eighteen HIV-seropositive individuals who did not complain of diarrhea were included in the study as a part of the control group. Ethical approval for the study was granted by the Ethics Committee of B.J. Government Medical College and Sassoon General Hospitals, Pune. Written informed consent was obtained from each patient prior to the sample collection.

Detailed history records were obtained and clinical examinations were done. The HIV infection was confirmed by three antibody detection tests: Combaids-RS (Span Diagnostics Ltd, Surat, India); Pareekshak HIV-1/2 Triline Card Test (Bhat Bio-Tech India Pvt. Ltd., Bangalore, India); and Instachk HIV 1+2 (One Step Anti-HIV [1 and 2] Tri-Line Test (Intec Products, Inc. P.R.C., Transasia Bio-Medicals Ltd., Mumbai, India). Freshly voided stool samples (at least two samples, on consecutive days) were collected from all the patients in the study. The samples were collected in clean, labeled, leak-proof, sterile, and wide-mouthed plastic containers and were transported immediately to the laboratory.

### Microscopy

All the samples were observed macroscopically for visible parasites, color, and consistency and presence of blood or mucus. The fresh fecal samples were examined for protozoa and helminths by direct wet mount examination using saline and iodine, as well as after concentration by formol-ether sedimentation concentration technique.

Direct smears of stool samples were prepared and stained with conventional Ziehl-Neelsen technique for detection of *mycobacteria*, the modified Ziehl-Neelsen stain [10] was used to detect coccidian parasites, and Gram stain was used to detect bacterial and fungal infections.

### Culture

For bacterial pathogens, the stool samples were inoculated on appropriate culture media and identified by the conventional standard biochemical tests [11]. The bacterial isolates were tested for antimicrobial susceptibility by the Kirby Bauer disc diffusion method according to the Clinical and Laboratory Standards Institute (CLSI) guidelines [12]. The testing was done with the following antibiotic discs obtained from HiMedia Laboratories, Mumbai: ampicillin (10 µg), gentamicin (10 µg), tetracycline (30 µg), chloramphenicol (30 µg), norfloxacin (10 µg), and furazolidone (50 µg). In cases where acid-fast bacilli were seen in the microscopic examination, the specimens were inoculated onto Lowenstein-Jensen medium after decontamination by modified Petroff’s method [11]. The specimens that showed fungal pseudohyphae on the saline mount and Gram stain were also inoculated onto Saborauds dextrose agar with antibiotics. Yeasts were identified by standard techniques [11].

### Data analysis

Demographic and other data were analyzed using the Statistical Package for Social Sciences (2007, SPSS version 16.0; IBM, Chicago, USA). The significant differences between categorical variables were determined using the chi-square test. A p-value of < 0.05 was considered statistically significant.

## Results

Among the 45 HIV-positive patients, 33 (73.33%) were males and 12 (26.67%) females. The ages ranged from 13 years to 62 years (mean 34.5 years) with maximum patients aged between 25 and 35 years (55.56%) followed by patients aged 35–45 years (26.67%). Of the 45 patients, 27 (60%) who presented with diarrhea, were studied primarily as the ‘study group,’ and 18 (40%) who presented without any complaints of diarrhea, were studied as the ‘control group.’ Other complaints of the patients included fever (53.33%), abdominal pain (42.22%), and vomiting (40%). The patients without diarrhea were admitted for various other conditions like tuberculous meningitis, cryptococcal meningitis, anemia, pulmonary tuberculosis, hepatitis, antiretroviral drug reactions, and generalized debility.

Thirty-six diarrheal pathogens were detected in the 45 patients in the study. Of these, 24 pathogens were identified in the patients with diarrhea, while 12 were identified in patients without diarrhea. For the purpose of the study, the isolates from the 'study group' were considered in the further assessment and evaluation.

Twenty-four pathogens were detected in the 27 diarrheal HIV-positive patients. The isolated pathogens were distributed as 14 parasites (14/24, 58.33% of all isolates), seven bacteria (7/24, 29.17% of all isolates), and three fungal isolates (3/24, 12.50% of all isolates) (Table 1).

**Table 1:** Pathogens detected from diarrheal patients (study group) and non-diarrheal patients (control group) in the study *Shigella flexneri, Mycobacterium tuberculosis *and *Candida albicans* were obtained as a mixed infection in one patient. *Isospora sp.* and *Cryptosporidium sp.* were obtained as a mixed infection in one patient. Mixed bacterial and parasitic infection was obtained in four patients. ETEC - Enterotoxigenic E. coli. EPEC - Enteropathogenic E. coli.

Pathogen	Diarrheal Patients *(Study Group)* (n = 27)	Patients without Diarrhea *(Control Group)* (n = 18)
Parasites	13 (48.15%)	6 (33.33%)
*Isospora sp.*	7 (25.92 %)	2 (11.11%)
*Cryptosporidium sp.*	4 (14.81 %)	2 (11.11%)
*Cyclospora sp.*	1 (3.7 %)	1 (5.56 %)
*Strongyloides stercoralis*	1 (3.7 %)	0
*Entamoeba histolytica*	1 (3.7 %)	1 (5.56 %)
Bacteria	7 (25.93%)	6 (33.33%)
*Escherichia coli – ETEC *	4 (14.81 %)	5 (27.78 %)
*Escherichia coli – EPEC*	1 (3.7 %)	1 (5.56 %)
*Shigella flexneri*	1 (3.7 %)	0
*Mycobacterium tuberculosis *	1 (3.7 %)	0
Fungi	3 (11.11%)	0
*Candida albicans*	3 (11.11 %)	0

Fourteen parasitic pathogens were identified in 13 patients of the study group (incidence: 13/27 = 48.15% patients). *Isospora sp. *was the most common parasite found in seven patients (7/27, 25.92%) followed by​ *Cryptosporidium sp.* in four patients (4/27, 14.81%). Of these, there was one case of mixed infection with *Isospora sp.* and *Cryptosporidium sp.* Other parasites that were detected included *Cyclospora sp.*, *E. histolytica *and *S. stercoralis* found in one patient each (1/27, 3.7%).

Bacteria were isolated from seven of the 27 diarrheal patients in the study group (7/27, 25.29%). The predominant isolate was *E. coli* isolated from five patients (5/27, 18.51%). The isolates were sent to the Central Research Institute (CRI), (Kasauli, Himachal Pradesh, India) for serotyping. ETEC was found to be the most common serotype (4/27, 14.81%) followed by EPEC (1/27, 3.7%), suggesting that 80% *E. coli* isolates were ETEC while 20% were EPEC. The other bacterial pathogens isolated included *Shigella flexneri* and *Mycobacterium tuberculosis *in one patient (1/27, 3.7%). They were found in the same patient as a mixed infection.

In the patients without diarrhea (control group*, *n=18), parasites and bacteria were detected in six patients each (6/18, 33.33%) (Table 1). The only bacteria isolated were *E. coli*, of which ETEC was in five patients (5/18, 27.78%) as compared to EPEC* *in one patient (1/27, 5.56%). *Isospora *and *Cryptosporidium* were detected equally in two patients each (2/18, 11.11%) followed by *Cyclospora* and *Entamoeba histolytica* in one patient each (1/18, 5.56%).

**Figure 1:**
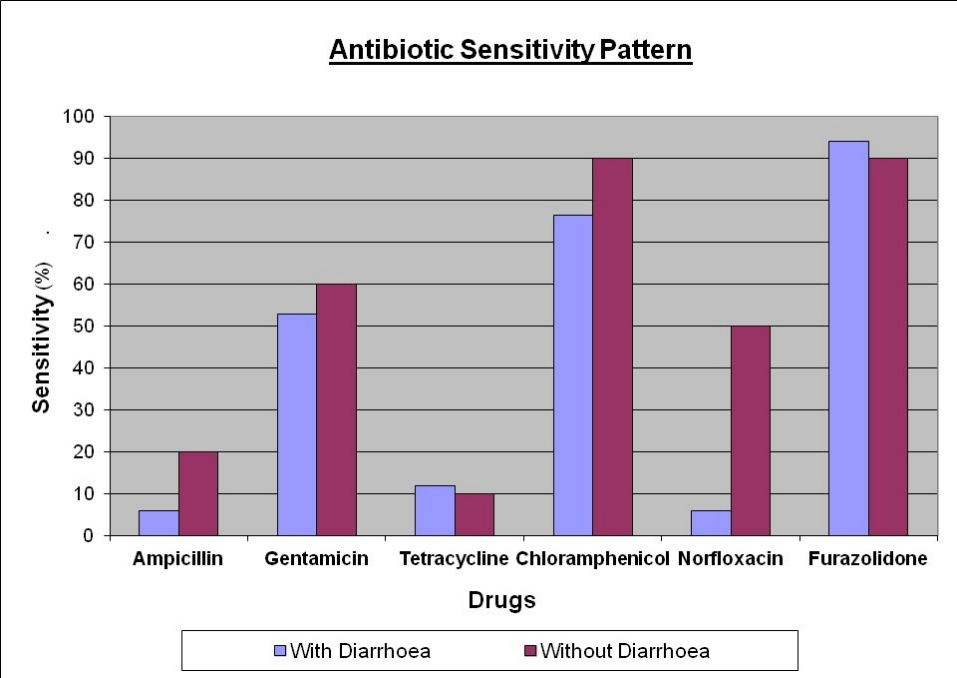
Antibiotic susceptibility testing of bacterial isolates

As evident from Figure 1, antibiotic sensitivity profiles revealed that most of the bacterial isolates from the patients with diarrhea (study group) were sensitive to furazolidone (94.11%), chloramphenicol (76.47%), and gentamicin (52.94%), while they were resistant to ampicillin (94.12%), norfloxacin (94.12%), and tetracycline (88.24%). The isolates from the patients without diarrhea (control group) showed sensitivity to furazolidone (90%), chloramphenicol (90%), gentamicin (60%), and norfloxacin (50%), while they showed resistance to tetracycline (90%) and ampicillin (80%). The diarrheal patients showed significant resistance to norfloxacin (sensitivity 5.88% vs. 50%, p < 0.05).

The range of the CD4 T cell count of the patients in the study was 15–565 cells/µL, mean 218.33 cells/µL. The mean CD4 count of patients with diarrhea (202.625 cells/µL) was lower as compared to non-diarrheal cases (239.28 cells/µL), though not statistically significant. As evident from Table 2, the maximum number of patients in the study group had CD4+ T cell count in the range of 100–200 cells/µL, below the AIDS-defining levels. The mean CD4 count in the patients with parasitic infection (195.47 cells/µL) was lower, though not statistically significant, than that of the patients without parasitic infection (214.16 cells/µL).

**Table 2:** Distribution of patients by CD4 count

CD4 Count Range (cells / µL)	Diarrheal Patients *(Study Group) *n = 27	Non–Diarrheal Patients *(Control Group*) n = 18
0 – 100	4	4
100 – 200	11	3
200 – 300	5	6
300 – 400	2	2
400 – 500	1	3
> 500	1	0

## Discussion

As HIV spreads, it impairs the immune cascade by affecting the CD4 T-lymphocytes, causing a marked drop in their number, while simultaneously multiplying in the host cells. The disequilibrium in the resistance of the host makes the host susceptible to a wide array of diseases, both opportunistic and non-opportunistic. The gastrointestinal tract is one of the most common sites affected in HIV-positive patients, with diarrhea being the commonest manifestation. Various pathogens are responsible for infecting the gut of HIV-positive patients. Most of them are opportunistic, which cause infection only when the immunity of the host falls below a critical level. These frequently cause fatal outcomes.

In the present study, 45 HIV-positive patients were studied. Of these, the maximum number of patients were in the age group of 25–35 years in both the sexes. The WHO has estimated that 0.8% (0.7–0.9%) of adults aged 15–49 years worldwide are living with HIV [13]. The Centers for Disease Control (CDC) attributed the high incidence in the youth to the high-risk behavior of the people in the age group, sexual promiscuity associated with this age, the rise in homosexuality, presence of other sexually transmitted diseases, and inadequate sex education [14].

In the present study, out of 27 HIV seropositive patients with diarrhea, parasites were detected in 13 (48.14 %) patients. Such high prevalence was also seen by Wiwanitkit, et al. who reported 50% incidence of parasitic infection amongst HIV-infected patients [15]. Table 3 shows the comparative incidence of parasitic infection in HIV-positive patients in various studies in India, reported in the literature.

**Table 3:** Comparative incidence of parasitic infection causing diarrhea in HIV-positive patients amongst various studies in the literature

	Abaver DT, et al. [16]	Nkenfou CN, et al. [17]	Fekadu S, et al. [18]	Present study
Incidence of parasitic infection causing diarrhea in HIV patients	22.7%	59.5%	47.8%	48.14%

Of the 19 patients who tested positive for parasites, 13 (68.4%) had diarrhea (study group). Hence, the isolation rate of parasites was more in the patients with diarrhea as compared to those without diarrhea. *Isospora *was the most commonly detected parasite. It was found in 25.92% patients with diarrhea. Mukhopadhya, et al. reported a lower prevalence for *Isospora* in 18% patients [3]. Prasad, et al. reported *Isospora* in 31% HIV patients with diarrhea, which is higher than in the present study [19]. There is a wide range of prevalence for *Isospora sp*. from 0.5–41% of HIV patients [20-21], probably due to the variation in the environmental conditions necessary for the survival and transmission of the protozoan parasite. Isosporiasis is rarely detected in the industrialized nations, the highest reported figure being three percent in USA [22]. *Cryptosporidium *was the next most common parasite isolated, it was found in 14.81% patients with diarrhea. Kumar, et al. reported them in 13.7% patients with diarrhea [23], similar to the prevalence in the present study. Tulli, et al. reported a very high prevalence of 39.8% for Cryptosporodial diarrhea in HIV patients [20], while Mukhopadhya, et al. reported *Cryptosporidium* in 10% of HIV patients [3]. Cryptosporidiosis* *has been reported from 6–37% of patients in various studies [24], outlining a wide range of prevalence of the organism. The simultaneous high prevalence of *Isospora* and *Cryptosporidium* is in agreement with the findings by Gupta, et al. who reported *Isospora *and *Cryptosporidium* in 41.1% and 20.6% patients with diarrhea, respectively [21]. Amongst the diarrheal patients, one patient showed a co-infection with *Isospora* and *Cryptosporidium*. The patient presented with profuse watery and mucoid diarrhea, associated with profound dehydration, vomiting, and fever. The simultaneous infection by both the pathogens may have led to the greater morbidity and the severity of symptoms in the patient. A similar mixed infection in one seropositive patient (0.82%) with diarrhea, with a similar stormy presentation, has been reported by Cranendonk, et al. [25].

Earlier studies from Northern India have found *Cryptosporidium *to be the most common parasite, while the prevalence of *Isospora *was found to be much lower [26-27]. Studies from Southern India, on the other hand, reported a higher prevalence of *Isospora belli* rather than *Cryptosporidium *[3, 23]. The present study is similar to the South Indian studies in reporting a higher incidence of isosporiasis. The regional variability of the parasites may be attributed to the variations in sources of drinking water, the level of sanitation, the number of asymptomatic carriers in the region and other environmental factors which may favor the persistence and transmission of a particular parasite species.

*Cyclospora* is a recently described emerging enteric pathogen in HIV-infected individuals. In the present study, *Cyclospora sp. *was detected in the stool of 3.7% patients with chronic diarrhea. Similar incidences of *Cyclospora* in the causation of chronic diarrhea in HIV-positive individuals have been reported from Chennai, South India [23] as well as from Jamnagar, West India [21].

The prevalence of non-opportunistic pathogens, viz. *E. histolytica *and *S. stercoralis *are highly variable. The prevalence of *E. histolytica *has been reported to be 1.6–11% [19, 26], while that of *S. stercoralis* had been reported to be 1.5-12% [28]. In the present study, *E. histolytica* and *S. stercoralis* have been reported in 3.7% patients each, in patients with diarrhea. The narrow range of prevalence reported for these parasites indicates that there is a uniformity of infecting rate of these pathogens as compared to the opportunistic pathogens whose incidence and prevalence is highly variable.

Amongst the patients without diarrhea (control group), *Isospora* and *Cryptosporidium* were the most frequent parasites detected, in 11.11% patients each. The lowered immunity in the gut of the HIV-positive patients allows the parasites to infect the gastrointestinal tract without producing symptoms. The high rates of these parasites in non-diarrheal patients indicate that the asymptomatic carriage of these organisms cannot be undermined, as these may eventually progress to clinical infections if left untreated for long. The actual rate of infection of these organisms in immunocompromised AIDS patients is likely to be underestimated due to the asymptomatic shedding of oocysts, and treatment with trimethoprim–sulfamethoxazole (co-trimoxazole) for other infections in AIDS cases may confer some protection against these parasites. However, the presence of *Isospora* and *Cryptosporidium* in HIV-positive cases with and also without diarrhea indicates an existing high risk of infection by these parasites.

Of the 27 patients in the study, bacteria were detected in 25.93% patients. Table 4 demonstrates the comparative incidence of bacterial infection in HIV-positive patients in various studies in the literature.

**Table 4:** Incidence of bacterial pathogenic infection in HIV-positive patients in various studies in India, reported in the literature

	Prasad KN, et al. (Lucknow) [19]	Mukhopadhya A, et al. (Vellore) [3]	Kownhar H, et al. (Madras) [8]	Present Study
Incidence of bacterial infection causing diarrhea in HIV patients	7.69%	19.67%	25%	25.93%

The most common bacterial isolate was *E. coli*  isolated in 18.51% patients, with the most common serotype being ETEC. A lower prevalence of *E. coli* (3.84%) has been reported by Prasad KN, et al. [19] However, there is a paucity of reports on the prevalence of bacteria in HIV patients in India. Also, there is inadequate data on the reporting of various serotypes of diarrheagenic* E. coli* common in HIV patients. Serotyping of *E. coli* isolates is important as the pathogenic mechanisms and the clinical illnesses produced by the different serotypes vary and so does the treatment of the infections produced by them. Hence, this subject needs further investigation and reporting. The commonest enteric bacterial pathogenic species encountered in the HIV-positive individuals are *Campylobacter*, *Salmonella, *and *Shigella flexneri *[3, 8, 19]; however, in the present study the three were found infrequently. In the present study, *Mycobacterium tuberculosis *was isolated from one patient (3.7%). The patient did not have pulmonary tuberculosis, suggesting primary abdominal tuberculosis. The gastrointestinal tract is an uncommon site for primary mycobacterialinfection. It is most commonly a part of disseminated disease in an immunocompromised host. Awole, et al. reported a prevalence of three percent for *mycobacterial sp.* in HIV patients with diarrhea [29]. The mycobacterial infection related with disseminated disease is associated with abdominal pain, generalized weakness, weight loss, and fever, as reported in the present study. The incidence of *Shigella species *as a causative organism for diarrhea is reported from 4–10% [7, 29]; while in the present study, it was isolated in 3.7% HIV-positive patients with diarrhea. *Mycobacterium tuberculosis, Shigella flexneri* and *Candida albicans *were isolated as a mixed infection from one patient. The patient had presented with chronic diarrhea since three months, associated with severe abdominal pain with guarding and rigidity, severe weight loss, dehydration, and fever. The patient died within 24 hours of admission. Multiple pathogen infections, although not frequently reported, is quite common, especially in our country where there is a widespread contamination of water and food sources with various pathogens.

Determination of antibiotic sensitivity testing revealed that the majority of the enteric bacterial isolates were sensitive to furazolidone, chloramphenicol, and gentamicin, but were relatively resistant to ampicillin, tetracycline, and norfloxacin. The isolates from diarrheal patients showed resistance to norfloxacin as compared to the non-diarrheal isolates. Norfloxacin is one of the drugs of choice for bacterial diarrhea in routine practice; however, the finding may indicate the emerging resistance of the bacterial isolates to the drug. The susceptibility of the isolates to furazolidone, chloramphenicol, and gentamicin may be of great value in the empiric management of diarrhea cases requiring antibiotic therapy in HIV patients. However, the present study only examined in vitro susceptibility patterns of the potential pathogens to antibiotics and such susceptibility patterns may not necessarily correlate with clinical usefulness.

*Microsporidia* were not detected in the present study. This may be due to the low fecal passage of the microsporidial oocysts and requirements of more specialized staining methods for their detection. Also, a high rate of detection is found in intestinal biopsies rather than in stool sample. Other pathogens reported in the literature but not found in the present study include *Giardia lamblia, Blastocystis hominis, helminths, Campylobacter jejuni, Salmonella spp, Enteroinvasive E. coli (EIEC), Aeromonas sp.* etc. Their nondetection could be due to the small sample size and the short duration of the study. Higher sampling may aid in the detection of these organisms.

In the present study, CD4+ T lymphocyte count was lower in diarrheal HIV patients (202.63 cells/µL) as compared to non-diarrheal ‘control group*’* patients (239.28 cells/µL), though the difference was not statistically significant. CD4+ T cells are critical in mounting an effective immune response to infections; hence, the decrease in local gut immunity due to a fall in CD4 T lymphocytes and the abnormality in their structure predispose the individual to the infection by intestinal pathogens. In an otherwise immunocompetent person, these pathogens may not produce clinical illness, but owing to the decreased resistance to infection, they produce mild to severe, and sometimes even fatal, disease in the immunocompromised hosts. The CD4 count in patients harboring parasites was 195.47 cells/µL, which is well below the WHO immunological criteria for severe HIV infection and AIDS, defined as CD4 count below 350 cells/µL [4]. Thus, people with AIDS are predisposed to suffering from an intestinal parasitic infection. Most people with parasitic or bacterial infection had CD4 in the range of 100–300 cells/µL. Hence, parasitic or bacterial pathogens should be looked for in patients with CD4 counts less than 300 cells/µL, especially as the pathogen may not produce clinical illness, but the person may just be an asymptomatic carrier or even a future patient. Wiwanitkit, et al. document that opportunistic intestinal pathogens were significantly more frequent in a low immunity group with diarrhea [15].

## Conclusions

The study emphasizes the role of opportunistic pathogens in the causation of diarrhea in HIV-positive patients. Thus, opportunistic intestinal parasitic or bacterial infection should be suspected in any HIV-infected patient with advanced disease, who may present with diarrhea. However, the importance of tropical, epidemic, non-opportunistic intestinal pathogenic infection amongst HIV-infected patients should not be neglected. An HIV epidemic can be more effectively managed if physicians and health planners are aware of this information. The prevalence of bacteria in HIV patients and their antibiotic susceptibility patterns should be kept in mind while initiating therapy against bacterial diarrhea, so as to avoid ineffective treatment and development of resistance.

The increased incidence of *Cryptosporidium* and *Isospora* in patients with and without diarrhea necessitates further characterization of their role as potential pathogens in HIV patients and prevailing high asymptomatic carrier states. Hence, stool examination of non-diarrheal HIV-positive patients can help in identifying carriers of parasites who are future patients. Prophylactic chemotherapy to these patients can be helpful. The CD4 T cell counts and their relation to the prevalence of various etiologic agents in a particular area should be considered before instituting empirical therapy to AIDS patients.

In a developing country like India, the magnitude of the enteric infection in HIV patients further adds to the existing financial burden of the disease. The patients usually are from a poor socio-economic background and they can hardly afford the cost of treatment. Therefore, it is suggested that steps be taken to prevent the occurrence of these diseases in AIDS patients, as often, the disease may take fulminant form.
